# Case Report: Disseminated *Talaromyces marneffei* Infection in a Patient With Chronic Mucocutaneous Candidiasis and a Novel STAT1 Gain-of-Function Mutation

**DOI:** 10.3389/fimmu.2021.682350

**Published:** 2021-08-05

**Authors:** Kuang Chen, Junfeng Tan, Shenxian Qian, Shenghai Wu, Qiong Chen

**Affiliations:** ^1^Department of Hematology, Affiliated Hangzhou First People’s Hospital, Zhejiang University School of Medicine, Hangzhou, China; ^2^Department of Laboratory Medicine, Affiliated Hangzhou First People’s Hospital, Zhejiang University School of Medicine, Hangzhou, China

**Keywords:** chronic mucocutaneous candidiasis, STAT1, gain-of-function mutation, *Talaromyces marneffei*, primary immune deficiency

## Abstract

Chronic mucocutaneous candidiasis (CMC) is a disorder of recurrent or persistent chronic noninvasive symptomatic infections of the skin, nails and mucous membranes. This disorder is primarily caused by *Candida albicans*. Many factors, including primary immunodeficiencies, can make a host more susceptible to CMC. Signal transducer and activator of transcription 1 (STAT1) gain-of-function (GOF) mutations are the most common genetic etiologies of CMC. We describe a case of CMC with disseminated *Talaromyces marneffei* infection caused by a new pathogenic Y287N mutation at amino acid 287 in the coiled-coiled domain of STAT1, which was identified using whole-exome sequencing. Position 287 might be a hot spot for missense mutations because several amino acid substitutions were found there. Flow cytometry suggested that the Y287N mutation might reduce the expression of IL-17 of Th17 cells in peripheral blood mononuclear cells stimulated by phorbol myristate acetate and ionomycin. The STAT1 Y287N GOF mutation may be the direct cause of recurrent cutaneous and mucosal candidiasis, including the *T. marneffei* infection in this patient.

## Introduction

Chronic mucocutaneous candidiasis (CMC) is classified as a primary immunodeficiency disease by the International Union of Immunological Societies Expert Committee for Primary Immunodeficiency in 2015 (1). It primarily presents as recurrent and persistent superficial infections with *Candida albicans*, affecting the mucous membranes, skin and nails (2, 3). CMC is associated with an impaired Th17 cell response caused by signal transducer and activator of transcription 1 (STAT1) gain-of-function (GOF) mutations. STAT1 GOF mutations, which occur in the functional coiled-coiled domain or the DNA-binding domain of STAT1, are the genetic etiology for CMC, and they can cause host susceptibility to bacteria, viruses, and intracellular bacteria addition to *Candida albicans (* 4). *Talaromyces marneffei* is an invasive pathogenic fungus, and *T. marneffei* infection often occurs in immunocompromised patients and patients with primary immunodeficiencies (5). However, *T. marneffei* infection is rare in primary immunodeficiency patients with candidiasis. In this report, we describe the clinical and genetic findings of a patient with CMC who also had disseminated *T. marneffei* infection due to the novel gain-of-function mutation Y287N in STAT1.

## Case Presentation

**Figure 1:**
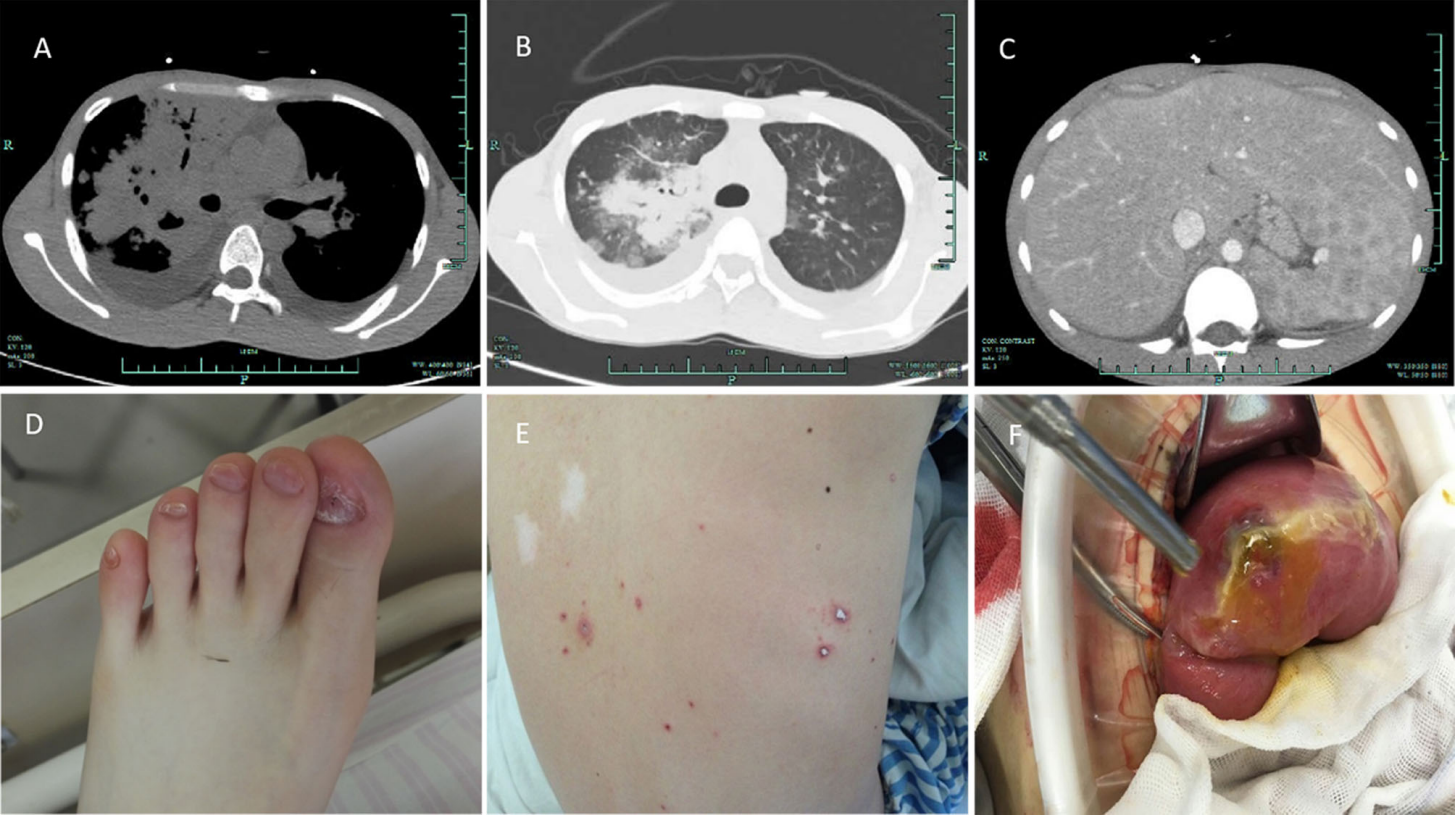
Clinical photos. **(A)** Bilateral lung infection with a bilateral pleural effusion, a paraspinal mass of the right lower mediastinum, and pericardial effusion. **(B)** CT of pulmonary Talaromyces marneffei infection. **(C)** Enlarged spleen, multiple splenic lesions, cholecystitis, and peritoneal effusion. **(D)** Abnormality of the patient's toenails. **(E)** Rash on the patient's back due to Talaromyces marneffei infection. **(F)** Surgical exploration demonstrated intestinal perforation and edema of the surrounding mucosa.

Three months after birth, the patient had recurrent mycotic stomatitis, tinea capitis and onychomycosis. He had a poor physique throughout childhood and often went to the hospital because of *Candida* dermatitis (**Figure 1D**). Because of long-term malnutrition, he was thin with a body mass index of 16.52. Knowing the medical history, it was found that he had no other obvious infection except *Candida* dermatitis. At the age of 20, he was admitted to the hospital with severe pneumonia. His body temperature reached a peak of 39.3°C. He had chills, cough and white sticky sputum but no night sweats, no chest tightness, no headache, and no abdominal pain or diarrhea. Pulmonary CT revealed bilateral pulmonary infection with bilateral pleural effusion, a patchy high-density shadow near the right lung hilum, a paraspinal mass in the right lower mediastinum, and pericardial effusion (**Figures 1A, B**). Fiberoptic bronchoscopy showed that the mucosal surface of each segment of the right bronchus was covered with a large amount of white caseous necrotic tissue. Acid-fast bacilli staining and testing for *Mycobacterium tuberculosis* DNA in bronchoalveolar lavage fluid were both negative. Sputum acid-fast staining was performed repeatedly during hospitalization, and all of the results were negative. During this hospitalization, routine bone marrow smears showed poor megakaryocyte function and macrophage phagocytosis of red blood cells, white blood cells and platelets. He was positive with IgG antibodies of *Rubella virus*, *Herpes virus*, and *Epstein-Barr virus* (**Table 1**). Upon physical examination, the patient was awake, thin, and had normal secondary sexual characteristics, chapped and brittle nails on his hands and feet, a thick white membrane on his tongue, no ulcers, and no obvious swelling of bilateral tonsils. No bleeding was observed on the skin or mucosa, and no rashes, no enlargement of the liver or spleen, no tenderness of the abdomen, no rebound pain in the abdomen, no palpation of superficial lymph nodes and normal auscultation of the heart and lungs were observed. After admission, empirical anti-infective treatment was performed with meropenem and voriconazole due to his unidentified pathogenic infection.

**Table 1:** Laboratory examination of specific antibodies and antigens.

Test items	Results	Unit	Reference intervals
TOX-IgG	0.10	kIU/L	<1.60
TOX-IgM	0.13	index	<0.50
RV-IgG	68.61	kIU/L	<5.00
RV-IgM	0.06	index	<1.20
CMV-IgG	228.78	kAU/L	<6.00
CMV-IgM	0.10	index	<0.85
HSVI-IgG	6.15	AU/mL	0.00-19.00
HSVI-IgM	0.01	AU/mL	0.00-6.00
HSVII-IgG	1.17	AU/mL	0.00-13.00
HSVII-IgM	0.01	AU/mL	0.00-6.00
EBV-VCA/IgG	Positive		<1:10
EBV-VCA/IgM	Negative		<1:10
EBV-EA/IgG	Negative		<1:10
EBV-NA/IgG	Negative		<1:10

TOX, Toxoplasma; RV, Rubella. virus; CMV, Cytomegalo. virus; HSV, Herpes. virus; EBV, Epstein-Barr virus; VCA, Viral capsid antigen; EA, Early antigen; NA, Nuclear antigen.

Upper abdominal computed tomography suggested that the spleen was enlarged with multiple low-density shadows, ascites and cholecystitis (**Figure 1C**). Echocardiography demonstrated moderate pericardial effusion. The patient actively cooperated with the physician to repeatedly perform etiological examination and microbial culture, and *T. marneffei* was cultured from peripheral blood, bone marrow and sputum. The physician prescribed voriconazole for antifungal treatment. After treatment, the patient’s body temperature was normal, and his cough improved.

However, two weeks after his admission, several scattered umbilical fossa-like red blisters began to appear on the patient’s back, which gradually spread to both the upper limbs and the chest (**Figure 1E**); the blisters were not itchy but were painful. At the same time, the patient had double lower abdominal pain with fever, tenderness and rebound tenderness. Abdominal upright plain film showed free gas under the diaphragm, and an exploratory laparotomy found that there was a perforation in the small intestine, 15 cm from the ileocecal, and local edema was obvious. Next, a surgical operation was performed with partial small bowel resection and small intestinal transverse colostomy. Intestinal perforation with erosion, ulcers and polypoid lesions could be seen during the operation (**Figure 1D**). Following surgery, amphotericin B liposomes were used for antifungal treatment. We considered that acute intestinal perforation and rash may have occurred as a breakthrough infection of *T. marneffei* due to the patient’s immune deficiency. After surgery and amphotericin treatment, the patient gradually recovered, the lung inflammation was absorbed, the skin rash and abdominal inflammation subsided completely, and the body temperature returned to normal. After 6 months of standard anti-*T. marneffei* treatment with amphotericin B, the patient recovered from *T. marneffei* infection, pneumonia, and intestinal lesions. The patient still undergoes regular follow- up in the outpatient clinic.

**Figure 2:**
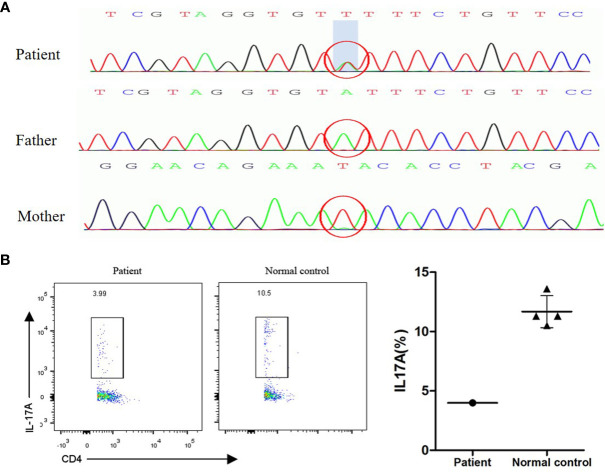
**(A)** STAT1 GOF mutation sequence in the patient and his parents. The patient had a heterozygous mutation in exon 10 of the STAT1 gene (Y287N, nt.859T > A). The parents had no mutations. **(B)** Th17 assay. Peripheral blood mononuclear cells from the patient and a control were isolated and stimulated with phorbol myristate acetate and ionomycin. Intracellular IL-17 was measured by flow cytometry.

Because the patient had recurrent or persistent infections of the skin, nails and mucous membranes with *Candida* spp., he could be diagnosed with CMC disease. CMC was especially common in patients with HIV infections before the introduction of effective antiretroviral treatments (2, 6); however, the laboratory tests for HIV, including for DNA and antibodies, were negative in our patient. From the literature, we know that mutations in some genes associated with primary immune deficiency(PID) can be responsible for susceptibility to CMC (6). Examples include STAT1 gain-of-function (GOF) mutations, STAT3 defects, and AIRE defects (3, 7). Clinical symptoms of CMC and persistent decreased lymphocytes led us to suspect that PID may be the cause of CMC in our patient; therefore, we used whole exome sequencing of the peripheral blood to explore the cause of PID and CMC. The results showed that there was a heterozygous mutation in exon 10 of the *STAT1* gene, leading to the substitution of the tyrosine amino acid at position 287 by asparagine (Y287N, nt.859T > A; the sequencing data can be obtained through accession number SRR13972765 on the National Center for Biotechnology Information website, **Figure 2A**). Human gene mutation database professionals indicated that a mutation at this site was a pathogenic mutation that was closely related to CMC (8). For example, a patient carrying a Y287H mutation began to develop CMC with bronchiectasis, dermatophytic infection and bacterial infections at 12 months (8). A patient carrying a Y287D mutation had thyroid dysfunction and bronchiectasis associated with dermatophytic infection, in addition to bacterial and viral infections at 3 months after birth (7). The Y287N substitution in STAT1 in our patient is the first time this mutation has been identified. There were no mutations in other genes, such as *AIRE, RFXANK, RFXAP, CIITA, EPG5, RFX5, IL17F, IL17RA, CARD9, CLEC7A, IL17A* or *IL17RC*.

In our study, the patient agreed to undergo the relevant examinations for immunodeficiency diseases after his recovery. Flow cytometric immunophenotyping revealed decreased total lymphocytes (200x10^6/L), total CD3+ T cells (119.6x10^6/L), total CD19+ B cells (76.3x10^6/L), total CD8+ T cells (51.6x10^6/L), total helper CD4+ T cells (62.3x10^6/L) and total CD16+/CD56+ NK cells (3x10^6/L) (**Table 2**). Autoantibodies and anti-IFN-γ antibodies were both negative. Peripheral blood mononuclear cell (PBMC) flow cytometry demonstrated that the expression of IL-17 decreased after PBMCs were stimulated by phorbol myristate acetate (PMA) and ionomycin (**Figure 2B**), which was consistent with the most common R274Q STAT1 mutation (9), suggesting that the Y287N STAT1 mutation might also affect the proliferation of Th17 cells and reduce the expression of IL-17. Y287N is a new substitution mutation; neither of the patient’s parents carried this mutation, indicating that it was a sporadic mutation.

**Table 2:** Laboratory examination of blood cells and immune cells.

Test items	Results		Unit	Reference intervals
White blood cell count	3.3	↓	10^9/L	3.5-9.5
Neutrophil count	3.1		10^9/L	1.8-6.3
Eosinophil count	0.00	↓	10^9/L	0.02-0.52
Lymphocyte count	0.2	↓	10^9/L	1.1-3.2
Hypersensitive C-reactive protein	54	↑	mg/L	0-8
Erythrocyte sedimentation rate	2		mm/h	0-15
Total lymphocyte count	200.0	↓	10^6/L	1200.0-3400.0
Total T lymphocytes CD3	119.6	↓	10^6/L	690.0-1760.0
Total B lymphocytes CD19	76.3	↓	10^6/L	90.0-323.0
Suppressor/cytotoxic T cell CD8	51.6	↓	10^6/L	190.0-658.0
Helper T cell CD4	62.3	↓	10^6/L	410.0-884.0
Natural killer cell CD16/56	3.0	↓	10^6/L	90.0-536.0
Total T lymphocytes CD3%	59.8		%	50.0-84.0
Total B lymphocytes CD19%	38.2	↑	%	5.0-18.0
Suppressor/cytotoxic T cell CD8%	25.8		%	15.0-44.0
Helper T cell CD4%	31.2		%	27.0-51.0
CD4/CD8 ratio	1.21			0.71-2.87
Natural killer cell CD16/56%	1.5	↓	%	7.0-40.0
CD3+CD25+ cell %	10.1		%	6.1-13.5
CD4+CD25+ cell %	9.7		%	3.3-10.6
CD3+HLA-DR+ cell %	24.7	↑	%	4.1-17.7
CD8+HLA-DR+ cell %	20.8	↑	%	1.3-13.4
CD8+CD38+ cell %	34.4	↑	%	2.3-15.0
IgG level	7.19	↓	g/L	7.23-16.85
IgM level	0.17	↓	g/L	0.63-2.77
IgA level	0.98		g/L	0.69-3.82
C3	0.88		g/L	0.85-1.93
C4	0.33		g/L	0.12-0.36

## Discussion

We reported a disseminated *T. marneffei* infection in a chronic mucocutaneous candidiasis patient with primary immunodeficiency caused by a STAT1 GOF mutation. CMC is a group of primary immunodeficiency disorders, and patients have different clinical manifestations, immunologic findings and genetic features depending on the immune status of the host (6–8, 10–12). Most patients have only persistent and recurrent superficial candidiasis, but some patients have additional infections with other fungi, viruses and bacteria (8, 13). CMC is also the most common infection occurring in autoimmune polyendocrinopathy-candidiasis-ectodermal dystrophy syndrome (APECED) (3, 14). The patient in our study presented with only recurrent and persistent infections of the skin, nails and mucous membranes, occurring since birth, and he did not have invasive candidiasis or autoimmune endocrine disease, which could be explained by his normal hormone levels. From the literature, we know that although *Candida albicans* is the most common species isolated from CMC patients, invasive species can also be found, including invasive *Cryptococcus* spp.*, Pneumocystis jirovecii, invasive Aspergillus* spp., and invasive *T. marneffei (* 8, 15). However, this patient did not suffer from invasive fungal infections and only had isolated instance of CMC before this hospitalization.

CMC can be acquired or caused by PID (3, 13, 16). Advances in genetic testing in the recent decade have expanded our knowledge of the immune mechanisms underlying CMC. Autosomal-dominant heterozygous missense STAT1 GOF mutations are the most common genetic cause of isolated CMC or CMC disease (4, 7). STAT1 GOF mutations influence the appropriate regulation of cellular responses to interferons, cytokines, growth factors and hormones and result in increased susceptibility to infection, immune dysregulation and malignancy (3). Toubiana et al. analyzed 274 CMC disease patients and found that most patients (98%) had STAT1 GOF mutations (8). Although STAT1 GOF mutations underlie a variety of infectious and autoimmune features, mucocutaneous fungal infection is the main manifestation, with a level of distribution that exceeds infections of the oral mucosa (93%), skin (57%), nails (56%) and esophageal/genital (56%) areas, while invasive fungal infections are rare, making up 10% of the total infections (8).

A number of STAT1 GOF mutations have been described. STAT1 GOF mutations increase STAT1 phosphorylation, resulting in STAT1-dependent increases in cytokines including IFN-α/β, IFN-γ, and IL-27 (4, 17). These cytokines influence the proliferation and differentiation of Th17 cells. We know that a decrease in Th17 cells directly causes IL-17 deficiency and affects IL-17-related regulatory pathways, which play an important role in the defense against *Candida*, bacteria and viruses (3). These pathways might be responsible for the long-term susceptibility to *Candida* infection observed in STAT1 GOF mutation patients. In our case, flow cytometry showed that the level of Th17 cells in the peripheral blood was lower than that of the normal reference when PBMCs were stimulated by PMA and ionomycin. Therefore, the STAT1 GOF mutation in this patient was probably the cause of his immunodeficiency and directly responsible for his recurrent cutaneous and mucosal candidiasis, as well as his *T. marneffei* infection.

The Y287N STAT1 GOF mutation carried by our patient is a new pathogenic mutation identified for the first time here. Two mutations with different amino acid substitutions were previously identified at the same site, Y287H and Y287D. Position 287 in STAT1 might be a hot spot for mutations.

The literature indicates that STAT1 GOF mutations can cause CMC, in addition to being responsible for host susceptibility to *T. marneffei* infections, especially in immunodeficient patients without HIV infection (18). Primary and secondary immunodeficiencies are risk factors for *T. marneffei* infection (5, 18). However, there have been few reports of *T. marneffei* infection in patients with primary immunodeficiency. Pan et al. reported that a child with primary immunodeficiency carrying a STAT3 mutation suffered from *T. marneffei* invasion of the intestinal system, which was characterized by multiple intestinal perforations, erosion, ulcers and polypoid lesions, among other symptoms of intestinal invasion (19).

*T. marneffei* infection is rare in CMC patients. In our study, the patient had a primary immune deficiency that was responsible for the susceptibility to *T. marneffei* infection. Although voriconazole was used empirically in the early stage of infection, the effect was not satisfactory. Breakthrough infection involving the lungs, skin, blood, bone marrow and intestinal tract occurred with *T. marneffei* indicating to the physician that there was bloodstream dissemination due to the patient’s immunodeficiency. After surgery and treatment with amphotericin B liposomes instead of voriconazole, the patient improved rapidly, indicating the necessity of using amphotericin B during the induction period.

Finally, we know that the patient’s hospitalization was caused by *T. marneffei* infection. However, he had a history of chronic mucocutaneous candidiasis from childhood, which prompted us to explore the cause of his immune deficiency. Fortunately, through whole exome sequencing, we confirmed that the patient carried a STAT1 mutation in the coiled-coiled domain, which might be responsible for his long-term susceptibility to *Candida* and *T. marneffei* infection.

In conclusion, we reported a disseminated *T. marneffei* infection in a chronic mucocutaneous candidiasis patient with primary immunodeficiency caused by a STAT1 GOF mutation. We identified a new pathogenic Y287N mutation in the coiled-coiled domain of STAT1 for the first time, which was the cause of PID with recurrent chronic mucocutaneous candidiasis and disseminated *T. marneffei* infection. Several amino acid substitutions have been identified at this site previously, and we hypothesize that this site may be a hot spot for mutation. Additionally, *T. marneffei* infection is no longer limited to patients with HIV infection; for non-HIV patients with *T. marneffei* infection, especially patients with recurrent immunodeficiency-related bacterial and fungal infection, the cause of their immune deficiency should be considered.

## Data Availability Statement

The datasets presented in this study can be found in online repositories. The names of the repository/repositories and accession number(s) can be found in the article/**Supplementary Material**.

## Ethics Statement

This study was approved by the Research Ethics Board of Hangzhou First People’s Hospital. The patients/participants provided their written informed consent to participate in this study. Written informed consent was obtained from the individual(s), and minor(s)’ legal guardian/next of kin, for the publication of any potentially identifiable images or data included in this article.

## Author Contributions

KC and QC wrote the manuscript. JT and SQ collected the patient’s medical records. SW and QC performed the laboratory tests. All authors contributed to the article and approved the submitted version.

## Funding

This work was supported by the Hangzhou Science and Technology Commission Social Development Project (20180533B31).

## Conflict of Interest

The authors declare that the research was conducted in the absence of any commercial or financial relationships that could be construed as a potential conflict of interest.

## Publisher’s Note

All claims expressed in this article are solely those of the authors and do not necessarily represent those of their affiliated organizations, or those of the publisher, the editors and the reviewers. Any product that may be evaluated in this article, or claim that may be made by its manufacturer, is not guaranteed or endorsed by the publisher.

## Supplementary Material

Supplementary Figure 1 The process of gating of flow cytometric. The levels of CD3+ cells and CD4+ cells were no obvious difference between patient and normal reference. Th17 cells secreting IL-17 showed decreased in the patient.
