# Supplementary material for: Case Report: Disseminated Talaromyces marneffei Infection in a Patient With Chronic Mucocutaneous Candidiasis and a Novel STAT1 Gain-of-Function Mutation
Source: Front Immunol. 2021 Aug 5;12:682350. doi: 10.3389/fimmu.2021.682350 (PMC8374937; doi:10.3389/fimmu.2021.682350)

**Supplementary material**

Figure S1 The process of gating of flow cytometric. The levels of CD3+ cells and CD4+ cells were no obvious difference between patient and normal reference. Th17 cells secreting IL-17 showed decreased in the patient.


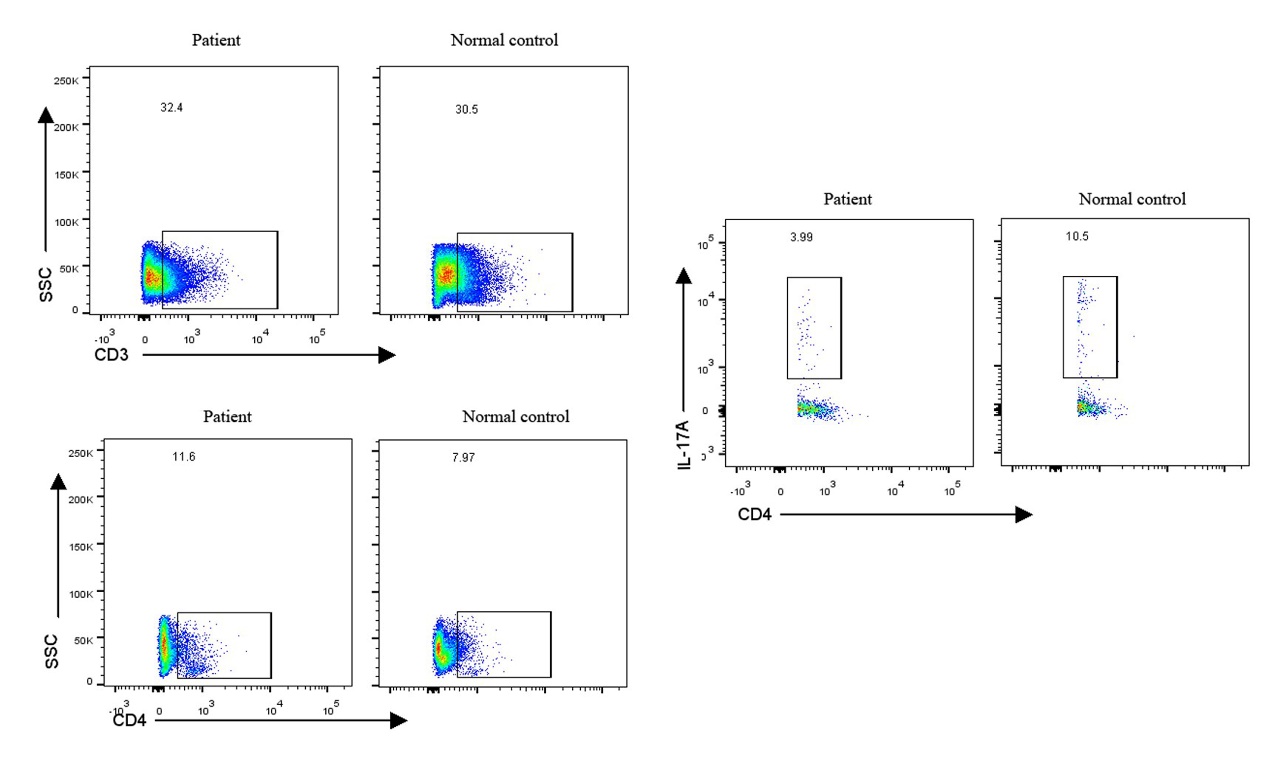

Supplement: Supplementary Figure 1 — The process of gating of flow cytometric. The levels of CD3+ cells and CD4+ cells were no obvious difference between patient and normal reference. Th17 cells secreting IL-17 showed decreased in the patient. [file DataSheet_1.docx]
